# Non-traumatic myositis ossificans

**DOI:** 10.4322/acr.2021.316

**Published:** 2021-08-20

**Authors:** Nidhin Rehman, Harish Sadashiva, Manoj Gopal Madakshira, Deep Kumar Raman

**Affiliations:** 1 Command Hospital, Department of Laboratory Medicine, Lucknow, Uttar Pradesh, India; 2 Command Hospital, Department of Oncosurgery, Lucknow, Uttar Pradesh, India

**Keywords:** Diagnostic Imaging, Myositis Ossificans, Pathology

## Abstract

Myositis ossificans (MO) is a benign, ossifying lesion that usually affects the skeletal muscle. The rare non-traumatic form of MO can cause diagnostic dilemma and management issues. These lesions, however, have similar radiology and histopathological characteristics described in the more frequently encountered traumatic forms. Depending on the stage of the lesion, the inherent feature of myositis ossificans varies, and so does the management of the lesion. We describe a non-traumatic MO occurring in latissimus dorsi of a young girl and discuss the review of literature on this rare subtype.

## INTRODUCTION

Myositis Ossificans (MO) is a benign, non-neoplastic, heterotopic ossifying process that is generally solitary, well encapsulated, and circumscribed.^1^ It is most commonly seen in the skeletal muscles but can also occur in tendons and subcutaneous fat.^2^ In 1924, Noble^3^ classified MO into three subtypes – (i) myositis (fibrous) ossificans progressiva, (ii) traumatic MO, and (iii) MO circumscripta without history of trauma. MO can develop at any age; however, it is most commonly seen in young, vigorous, athletic active adolescents, and adults.^1^^,^ 4 MO has characteristic radiological and histological features, which are best appreciated in the mature forms of this pseudo-tumour.^5^ We report a case of myositis ossificans in a 12-year-old girl who did not have any history of preceding trauma and review the current literature on non-traumatic forms.

## CASE REPORT

A 12-year-old girl presented with history of painful swelling in the right axilla for five days, with otherwise preserved general-condition. There was no history of antecedent trauma or injury. The girl was born following a full term normal vaginal delivery. The parents denied any injury during birth. On examination, a 10x8 cm sized, oval shaped, smooth surfaced firm to hard, tender swelling was seen originating from the right axilla extending from the anterior axillary to posterior axillary lines. The overlying skin was erythematous, and the lesion was fixed to the underlying muscle. The right shoulder movements were painful and terminally restricted. A working diagnosis of an abscess was made, and she was started on broad-spectrum antibiotics and anti-inflammatory medications. However, after five days no relief of symptoms was achieved. An ultrasonography of the axilla revealed a 6.5x4.2x4.1 cm well-defined encapsulated soft tissue lesion isoechoic to and likely originating from the muscle, without any increase in vascularity.

**Figure 1:**
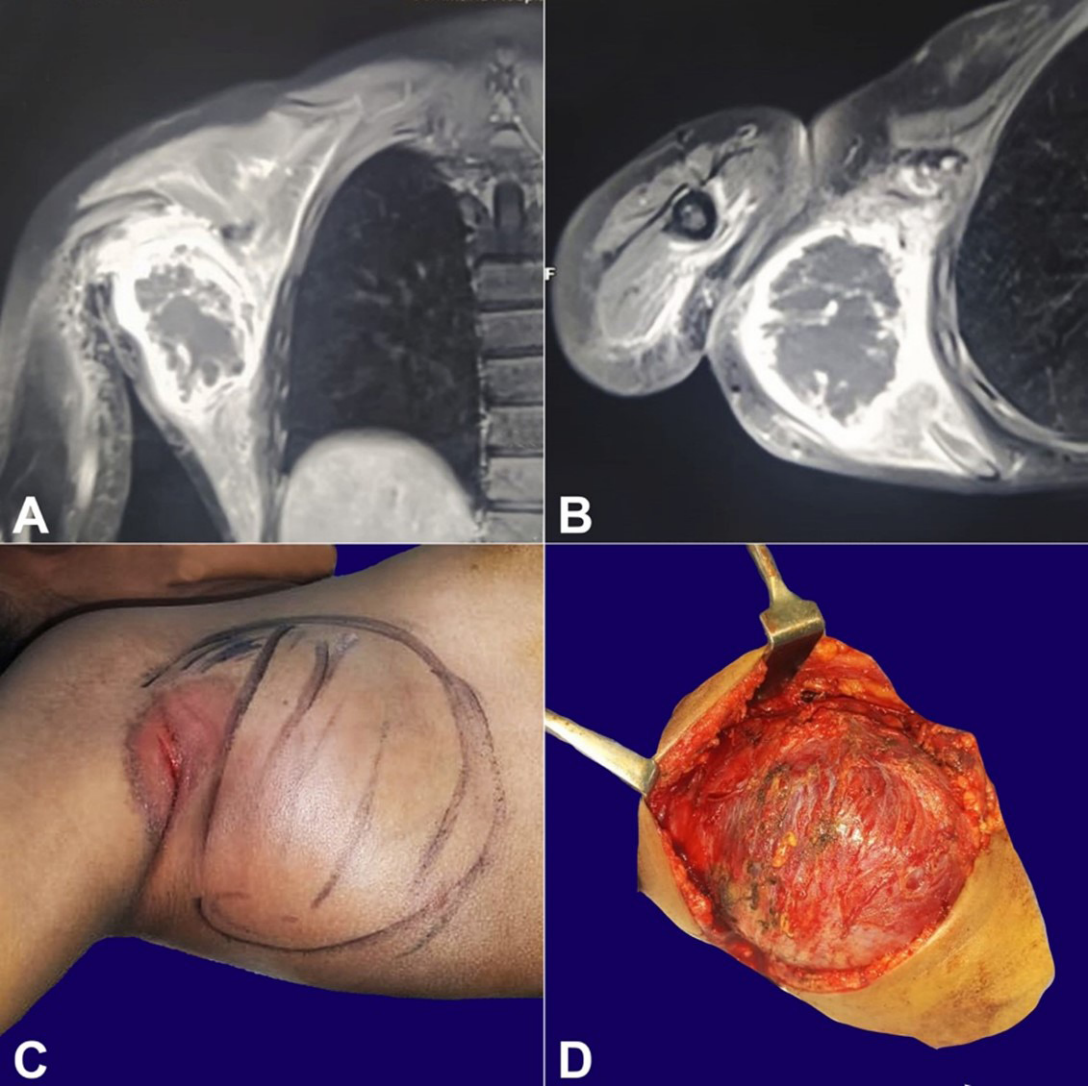
**A** and **B** – computed tomography of the shoulder; **A** – coronal plane; and **B** ­– axilla plane, image delineating the lesion with curvilinear peripheral ossification and central lucency; **C** and **D** – Pre-operative and intra-operative images of the lesion.

The magnetic resonance imaging revealed a well-encapsulated lesion within the belly of the latissimus dorsi muscle measuring 6.7x5.9x5.7 cm, which was homogeneously hypointense on T1 weighted images and hyperintense on T2 weighted images with some variegations of signal intensity due to the presence of numerous thin hypointense reticulations. On the post-contrast images, an enhancement was noted in the lesion’s capsule and in the septa that spanned throughout the stroma. No restriction of diffusion was noted. There was edema in the muscles of the right shoulder girdle and in the intermuscular plane. The computed tomography showed a well-defined lesion with peripheral calcification. (Figure 1A, B) A core biopsy was done and was suggestive of a myxoma.

The patient was submitted to exploratory surgery. Intraoperatively, the lesion arose from the latissimus dorsi muscle along the entire length from the origin to insertion. The planes of dissection were hardly achieved due to the dense fibrous adhesions with the surrounding skeletal muscle. A wide local excision of the lesion was done with a complete resection of the latissimus dorsi followed by primary closure of the defect (Figure 1C, D).

**Figure 2:**
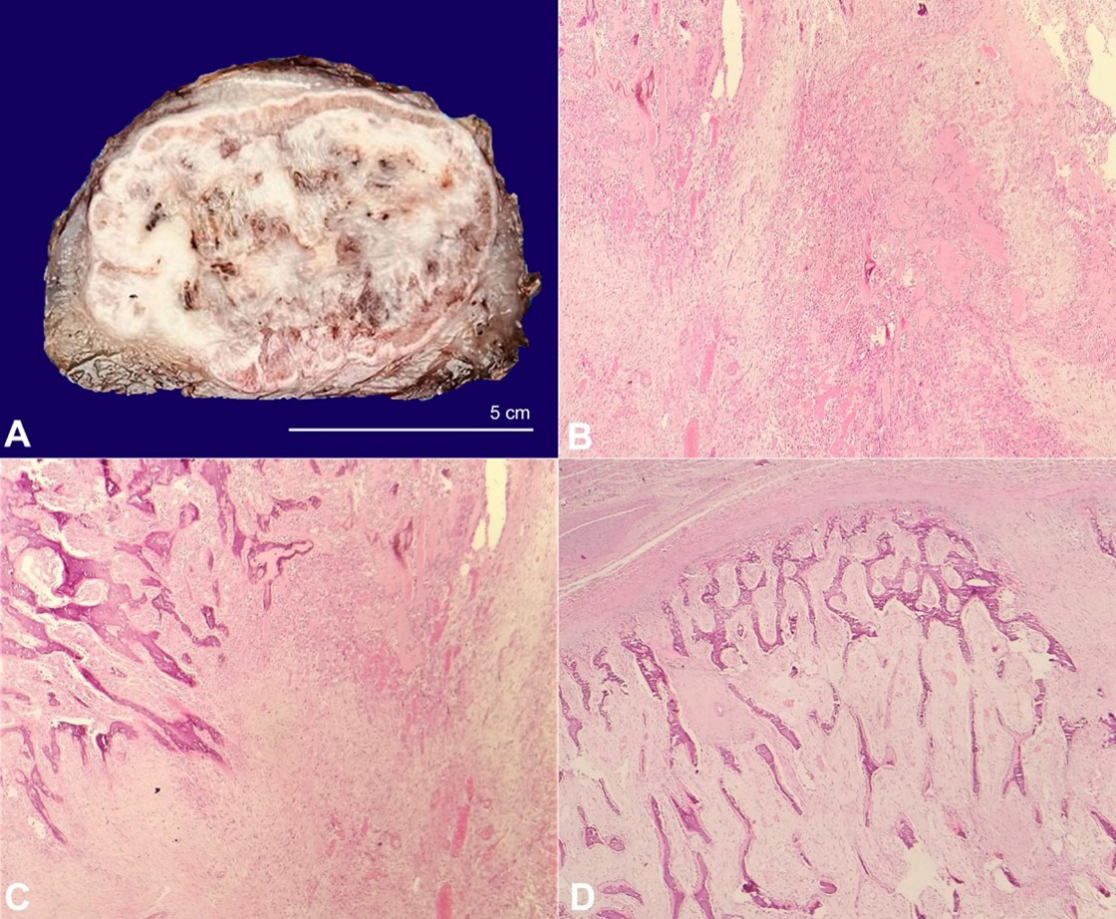
**A** – Gross view of the surgical specimen cut surface showing a well-circumscribed encapsulated lesion showing the pathognomonic zonation. **B**, **C** and **D** – Hematoxylin and Eosin-stained photomicrographs (40x magnification) of the tumor; **B** – The central zone shows hypocellular and hypercellular areas of loosely textured myofibroblasts with Interspersed dilated thin-walled capillaries; **C** – The intermediate zone shows the transition to ossification in the form of lace-like osteoid undergoing calcification; **D** – The outer zone shows organization of the calcified osteoid into a compact lamellar bone, which is separated from the surrounding tissue by a thick fibrous capsule.

The specimen was globular, measuring 10 x 8 x 4.5 cm. The cut section showed a well-encapsulated lesion with adequate surgical margins, the nearest of 0,7 cm. The periphery of the lesion showed bone formation, while the central area showed myxoid regions with few hemorrhagic foci (Figure 2A). The microscopy revealed a well-circumscribed encapsulated lesion with a characteristic zonation pattern. The innermost central zone was composed of loosely textured myxoid and richly vascular tissue composed of spindle to stellate myofibroblasts (Figure 2B). Intermingles were many lymphocytes, plasma cells, macrophages, and multinucleate foreign body type of giant cells along with fibrinous material. The intermediate zone showed an admixture of fibroblasts with osteoblasts, which were associated with paint-like eosinophilic homogenous osteoid that was separated by thin-walled ectatic capillaries (Figure 2C). The peripheral zone showed osteoid undergoing calcification with the formation of mature lamellar bony trabeculae (Figure 2D).

**Figure 3:**
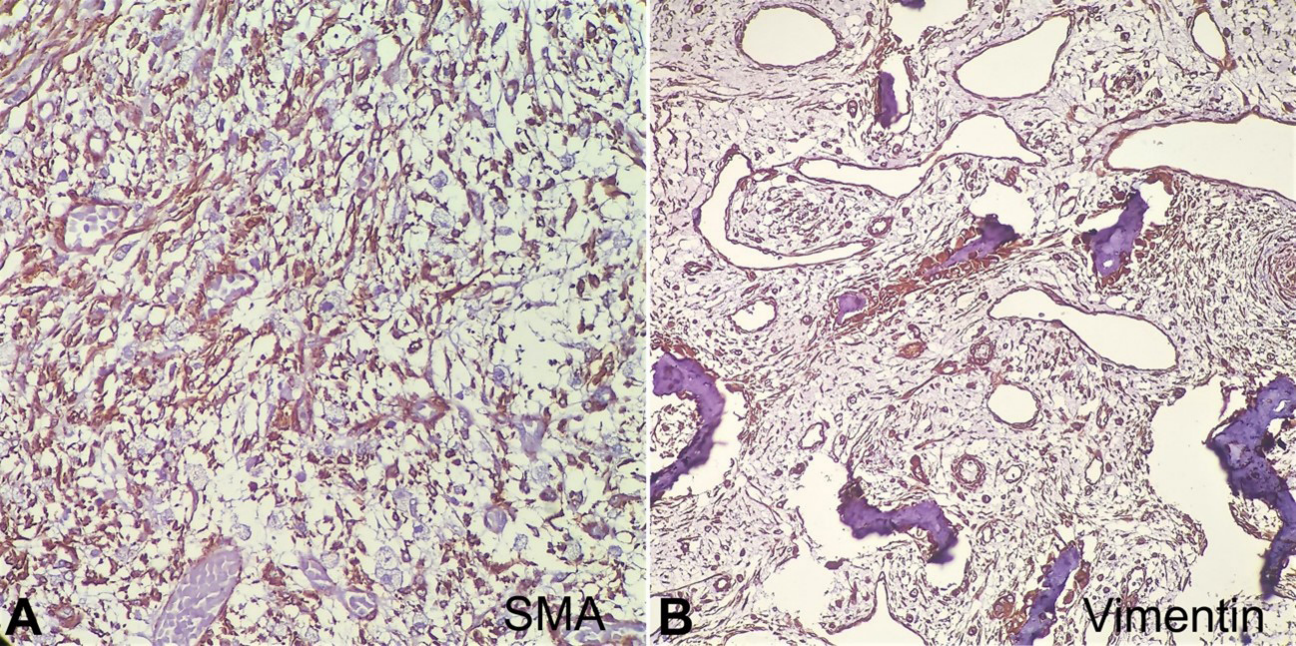
Photomicrographs (100x magnification) of the tumor. **A** – Smooth muscle actin (Clone EP188, Pathnsitu) highlights the myofibroblasts; **B** – Vimentin (Clone EP21, Pathnsitu) highlights the osteoblastic rimming of the bony trabeculae.

These trabeculae were rimmed by osteoblasts. The myofibroblasts were delineated by Actin, and the osteoblasts were better highlighted by Vimentin. (Figure 3A, B) The capsule was formed by loose myxoid to compressed fibrous tissue, separating the lesion from the surrounding skeletal muscle bundles. There was no nuclear pleomorphism or atypical mitosis. The histology was pathognomonic of Myositis Ossificans. The girl at 3 months follow-up is asymptomatic.

## DISCUSSION

MO verbatim is a term used to describe ossification secondary to inflammation of the muscle, which is also defined as a solitary, benign, ossifying soft tissue lesion manifesting within the skeletal muscle.^1^ The classification proposed by Noble^3^ described three entities: - (i) MO progressive, (ii) traumatic MO circumscripta, and (iii) non-traumatic MO cirumscripta.^1^^,^ 3 Traumatic MO is the most common subtype.^1^ MO progressive, also known as progressive fibrodysplasia ossificans is characterized by disabling fibrosis and ossification of muscles, which is caused by a mostly fatal, rare, autosomal dominant inherited disorder involving the Bone morphogenic protein receptor 1 (BMP1), affecting children below the age of 5 years.^6^ Non-traumatic circumscripta MO is the other rare condition, in which there is no antecedent history of trauma, as described in the index case.^7^

A PubMed search for the terms “Non-traumatic myositis ossificans” retrieved only anecdotal case reports in the English literature (Table 1).

**Table 1:** Review of literature with respect to non-traumatic myositis ossificans circumscripta

**Author**	**Age / Gender**	**Site/Muscle**	**Size cm**	**Symptoms**	**Imaging**	**Laboratory investigations**	**Treatment / Remarks**
Taylor^19^	40y/F	Right calf muscles	-	Painful lump	Calcification on X ray	-	Excision and primary repair
Saussez^17^	41y/M	Rhomboideus and Serratus anterior	7x 3	-	Calcification on CT;	-	Excision and primary repair
					Heterogenous T2WI on MRI		
Nishio^8^	83y/F	Left vastus medialis	6x5	-	Heterogenous T2WI,	-	Spontaneous regression in 2 months.
					Isointense T1WI on MRI;		
					Peripheral calcification on CT		
Juneja^11^	6y/M	Bilateral vastus intermedius, vastus lateralis and iliopsoas	-	Pain and restriction of movement	Peripheral calcification on CT	Case of cerebral palsy with spasticity	Remission following NSAIDs and physiotherapy
Jung^15^	42y/M	Paravertebral muscle, left	-	Pain in low back	Heterogenous T2WI,	-	Spontaneous remission
					Isointense T1WI on MRI;		
					Peripheral calcification on CT		
Abdallah^18^	31y/M	Left Paravertebral muscle L4-5	2x2	Low back ache	Peripheral calcification on CT	-	Excision
Bardouni^7^	27y/F	Posterior muscle compartment of left thigh	8x7	Painful swelling	Hyperintense T2WI,	ESR 31 mm/h, CRP 63 mg/dl, ALK Phosphatase 90U/L, Creatinine phosphokinase 200U/L	Radiotherapy 600cGy in six fractions. Reduction in size. Followed by excision
					Isointense T1WI on MRI;		
					Peripheral calcification on CT		
Mahale^14^	18y/M	Gluteus muscle	6x4	Pain and restriction of movement	Hyperintense T2WI,	ESR 30 mm/h	Excision
					Isointense T1WI on MRI;		
					Peripheral calcification on CT		
Li^20^	9y/M	Elbow	7x 6	Pain and restriction of movement	X ray – peripheral calcification	Leukocytosis 14000/mm^3^, CRP 13.66 mm/L, Normal Alkaline phosphatase	Excision
Simmonds^9^	5m/F	Right trapezius	2x2	Incidental	Hyperintense T2WI,	-	Excision
					Isointense T1WI on MRI;		
Ozbayark^16^	31Y/M	Left paraspinal muscle	-	Back pain	Peripheral Calcification on CT;	-	Excision
					Isointense on T1Wi and hyperintense on T2WI on MRI,		
					Intense uptake on PET		
Lee^13^	59y/F	Vastus lateralis	6x3	Pain	Checkerboard pattern on ultrasonography,	-	Spontaneous Remission
					Hypointense on T1WI		
					Hyperintense on T2WI;		
					Peripheral curvilinear calcification on CT		
Sferopoulos^2^	14y/M	Intercostal muscle	4x3	-	Peripheral calcification on CT	-	Excision
Igrutinovic^21^	75y/F	Biceps femoris	-	-	Peripheral calcification on CT	-	Excision with primary wound reconstruction and drainage
Kougias^12^	5y/F	Gluteus medius, both	-	Pain and restriction of movements	Peripheral calcification on CT	Adenovirus type 3 infection preceding immobilization	NSAIDs and physiotherapy, remission
	5 year	Gluteus muscle	-	Pain and restriction of movements	Peripheral calcification on CT	Staphylococcal infection preceding immobilization	NSAIDs and physiotherapy. Remission
Patru^22^	40 year	Forearm	-	Weakness	Peripheral calcification on CT	-	Excision
Index case	12 year	Latissimus dorsi	10x 8	Pain and swelling	Hypointense on T1WI	-	Excision
					Hyperintense on T2WI;		
					Peripheral calcification on CT		

Cm= centimeter; F= female; m= month; M=male; Y=year, CT=computed tomography, MRI=magnetic resonance imaging, T1WI= T1 weighted image, T2W1=T2 weighted image.

Non-traumatic MO has been reported in a wide age range from as young as 5 months to as old as 83 years.^8^^,^ 9 This lesion does not show any gender predilection. Various muscle groups have been described to be affected, with the thigh and hip compartment being the commonest.^7^^,^ 8 ^,^ 10 ^-^ 14 Isolated reports of rarer muscle groups such as the paraspinal, trapezius, rhomboids, teres minor, and intercostal muscle have also been reported.^2^^,^ 9 ^,^ 15 ^-^ 17 The index case was seen to arise from the latissimus dorsi, which has not been previously reported. This finding delineates that non-traumatic sub-type of MO does not show any predisposition towards any specific muscle. The size of the lesion varies depending on the site, ranging from 2 to 8 cm in the largest dimension.^7^^,^ 18

Some of the patients of non-traumatic MO had an underlying or antecedent illness such as viral and bacterial infections or spasticity due to cerebral palsy.^11^^,^ 12 The cause for developing MO begins with injury to the muscle, which sets in motion an inflammatory cascade. The cesspool of cytokines such as Transforming Growth Factor and bone morphogenic proteins target the endothelial cells in the muscle. These activated endothelial cells undergo an epithelial-mesenchymal transition to form the pluripotent mesenchymal stem cells. These stem cells differentiate to form the myofibroblasts, chondrocytes, and osteoblasts, culminating in bone formation.^1^ MO goes through 3 phases of maturation.^1^ The maturation beings with a poorly circumscribed area composed of densely packed immature mesenchymal cells, which is followed by an intermediate stage with endochondral ossification, evidenced by the presence of peripheral calcification radiologically. The final stage is the mature stage, which is characterized by the peripheral zone of compact lamellar bone and central loose myofibroblasts separated by endochondral ossification. Throughout the three stages, the patients usually complain of pain and swelling accompanied by restriction of movement if the lesion is present in the vicinity of a joint.^1^ The pain gradually improves as the lesion matures.

The imaging diagnosis of MO is better achieved by computed tomography, especially in the mature stage.^5^ Lee at al.^13^ have described a ‘checkerboard pattern’ on ultrasonography, in the early lesion of MO. Magnetic resonance imaging usually shows isointense to hypointensity on T1 weighted images and heterogenous intensity on T2 weighted images.^1^ Computed tomography is diagnostic, with the presence of the peripheral curvilinear calcific rim and central lucence.^1^^,^ 5 As reported in conventional traumatic MO, some cases of non-traumatic MO have also reported concurrent raised erythrocyte sedimentation rate and rise in serum levels of C-reactive protein, alkaline phosphatase, and creatinine phosphokinase.^7^^,^ 13 ^,^ 14

In most cases, the diagnosis is established after a biopsy. The gross specimen of MO usually shows a well-circumscribed lesion that has a gritty sensation on sectioning due to the calcification. The cut surface shows a white, soft and gelatinous or hemorrhagic center and yellowish-grey granular surface in the periphery. The histology is characterized by the zonal patterns, which reflect different degrees of cellular maturation that are usually conspicuous after 3 weeks.^1^^,^ 2 In the mature stage, the central zone will be composed of immature, loosely packed, richly vascular myofibroblastic tissue. A trucut biopsy from this area alone, can result in an erroneous report of nodular fasciitis, myxoma or mere granulation tissue.^1^ These myofibroblasts can show mild degrees of nuclear pleomorphism and increased mitosis. However, nuclear hyperchromasia and atypical mitosis are seldom seen.^1^ Also seen part of this milieu are varying number of macrophages, lymphocytes, plasma cells, fibrinous material, and multinucleated giant cells. In addition, there is prominent endothelial proliferation, focal areas of hemorrhage, and entrapped atrophic or necrotic muscle fibers. The intermediate zone, which surrounds the central zone, shows cellular condensation into poorly defined trabeculae with a mixture of fibroblast and osteoblasts separated by thin-walled ectatic vascular channels. While moving towards the periphery, the osteoid undergoes increasingly endochondral ossification, which finally evolves into mature lamellar bone.^1^ Characteristically, the bone formation is most prominent at the periphery and margins of the lesion, where they are rimmed by a monolayer of osteoblasts which are monotonous and have very little variation in size and shape.

The differential diagnosis in the mature stage, include periosteal reaction, para-osteal osteochondromatosis, and sinister extra-skeletal and para-osteal osteosarcoma.^1^ The prognosis of MO is excellent considering that, in many instances, it shows as a self-limiting process, with reports of spontaneous remission.^8^^,^ 15 Early stages of non-traumatic MO have been successfully treated by non-steroidal anti-inflammatory drugs and physiotherapy.^11^^,^ 12 An anecdotal report showed a considerable decrease of the lesion’s size following localized radiotherapy.^7^ However, all cases of mature non-traumatic MO need surgical management for effective resolution by excision and primary closure.^1^

In conclusion, a possibility of non-traumatic MO needs to be considered when a patient presents with a painful lump arising in a muscle showing the presence of calcification on radiology. Though the lesion remains well circumscribed, depending on the stage of the lesion, the degree of calcification and mature bone formation varies. Biopsy of the lesion is diagnostic in identifying the characteristic zonation pattern, circumscribed nature, and lack of nuclear pleomorphism helps to negate sinister possibilities. Management of mature lesions mandates surgical excision. However, early stages can be effectively managed by NSAIDs and physiotherapy alone.
